# Supplementary material for: Effects of Synthetic Toll-Like Receptor 9 Ligand Molecules on Pulpal Immunomodulatory Response and Repair after Injuries
Source: Biomolecules. 2024 Aug 1;14(8):931. doi: 10.3390/biom14080931 (PMC11353191; doi:10.3390/biom14080931)
Supplement: Supplementary file 1 [file biomolecules-14-00931-s001.zip › biomolecules-3094805-supplementary/Table S1.pdf]

Table S1. The distribution of animals per experimental group.

Experimental solution	Concentration	Observation period	Number of animals	Number of teeth (n)
DW	–	1w	5	10
		2w	6	12
HBSS	1x	1w	6	12
		2w	6	12
CpG-ODN A	0.63 mg/ml (0.1 mM)	1w	9	18
		2w	11	22
	5 mg/ml (0.8 mM)	1w	6	13
		2w	7	14
CpG-ODN B	0.63 mg/ml (0.1 mM)	1w	10	20
		2w	11	22
	5 mg/ml (0.8 mM)	1w	7	14
		2w	8	16
