# Supplementary figures and images for: Effects of Synthetic Toll-Like Receptor 9 Ligand Molecules on Pulpal Immunomodulatory Response and Repair after Injuries
Source: Biomolecules. 2024 Aug 1;14(8):931. doi: 10.3390/biom14080931 (PMC11353191; doi:10.3390/biom14080931)

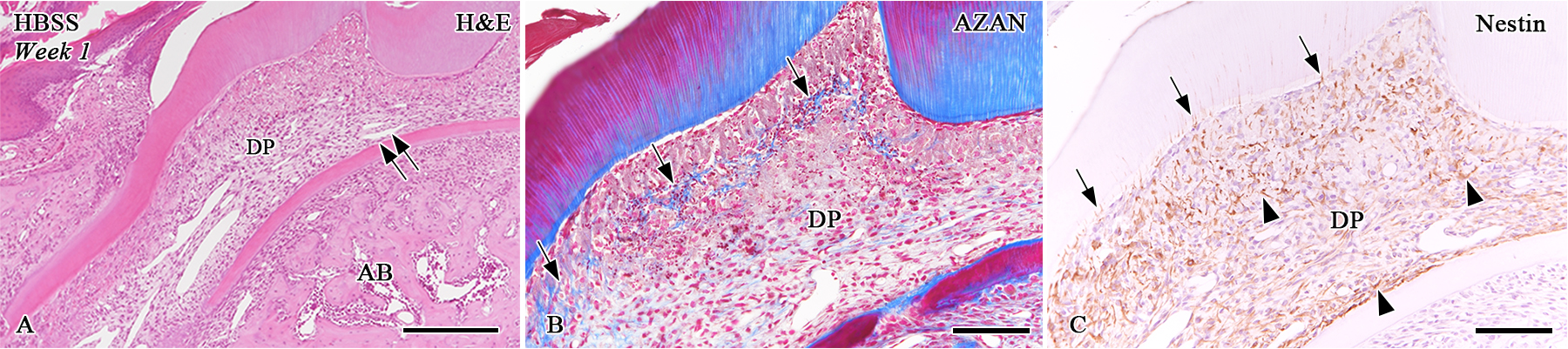

Supplement: Supplementary file 1 [file biomolecules-14-00931-s001.zip › biomolecules-3094805-supplementary/Suppl 1 - HBSS Week 1 - New.jpg]

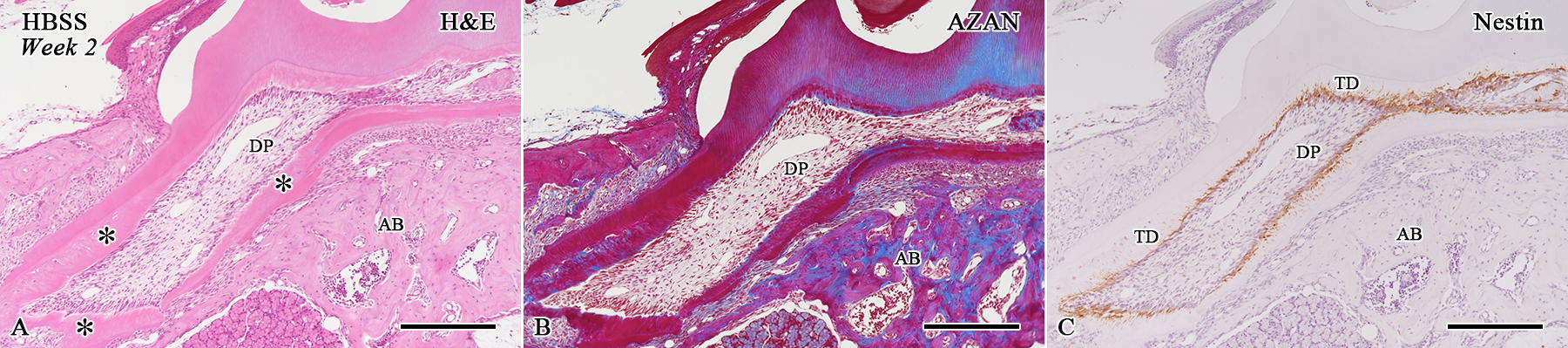

Supplement: Supplementary file 1 [file biomolecules-14-00931-s001.zip › biomolecules-3094805-supplementary/Suppl 2 -HBSS Week 2 - New.jpg]

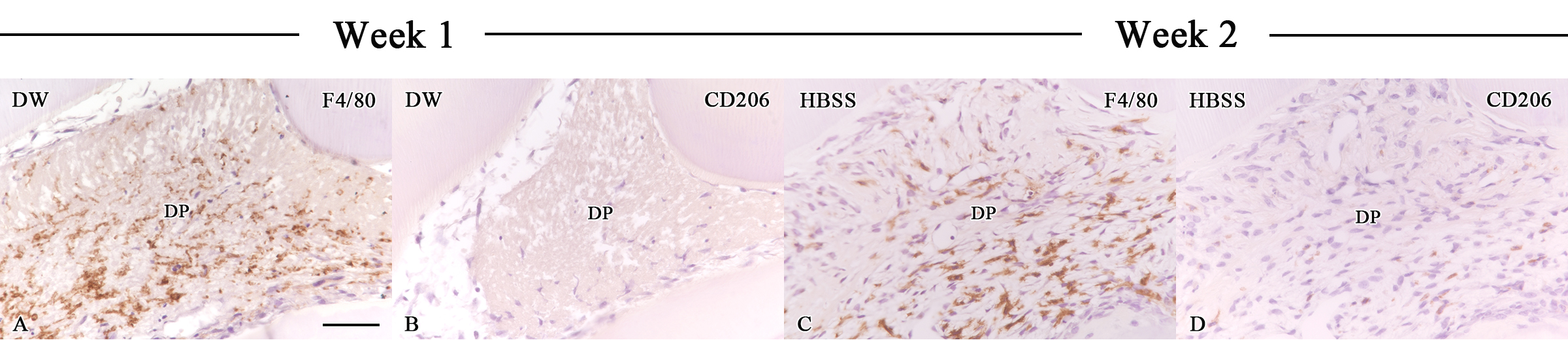

Supplement: Supplementary file 1 [file biomolecules-14-00931-s001.zip › biomolecules-3094805-supplementary/Suppl 3- F480 CD206 Weeks 1-2.jpg]

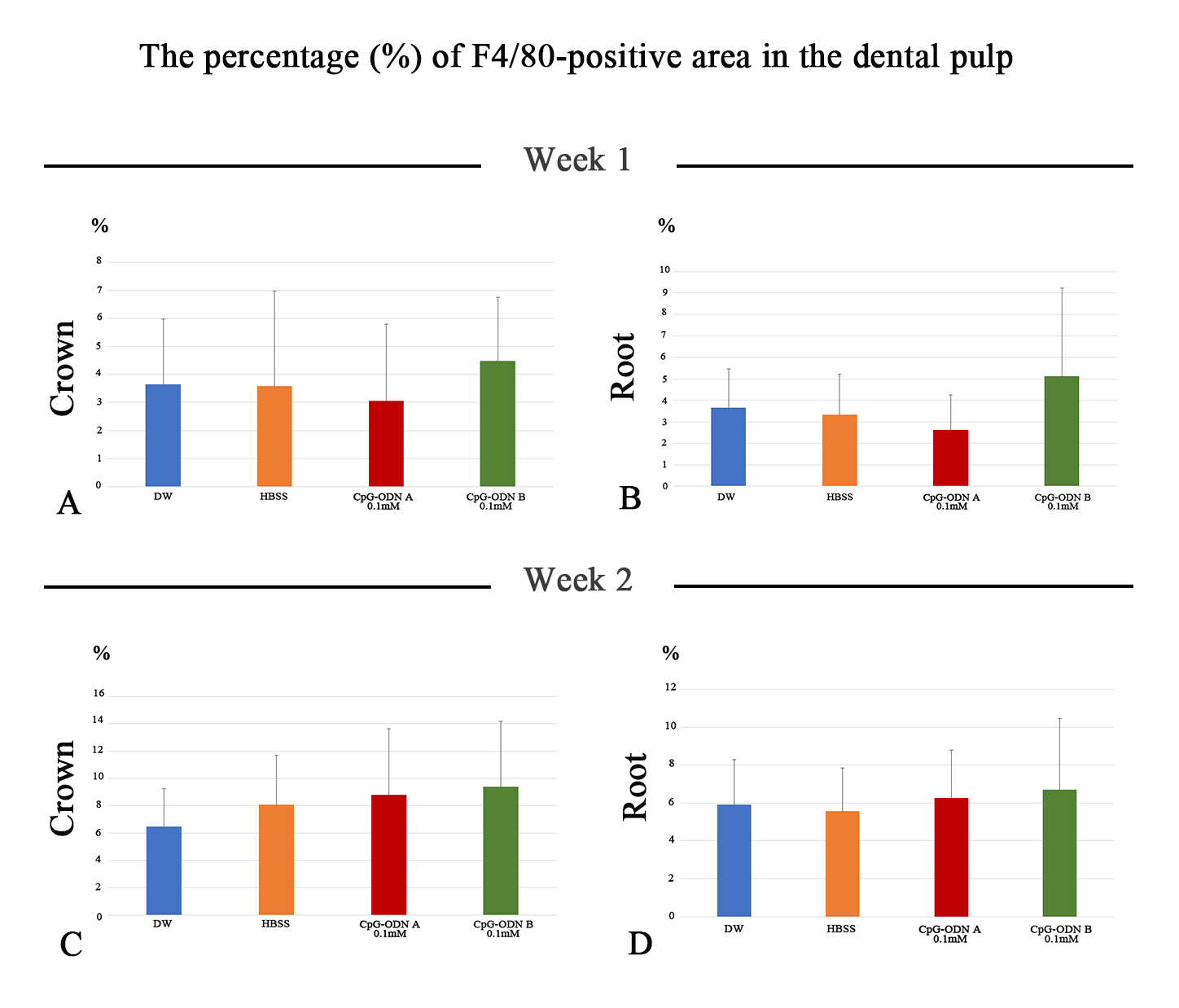

Supplement: Supplementary file 1 [file biomolecules-14-00931-s001.zip › biomolecules-3094805-supplementary/Suppl 4 F4-80 graph plate.jpg]

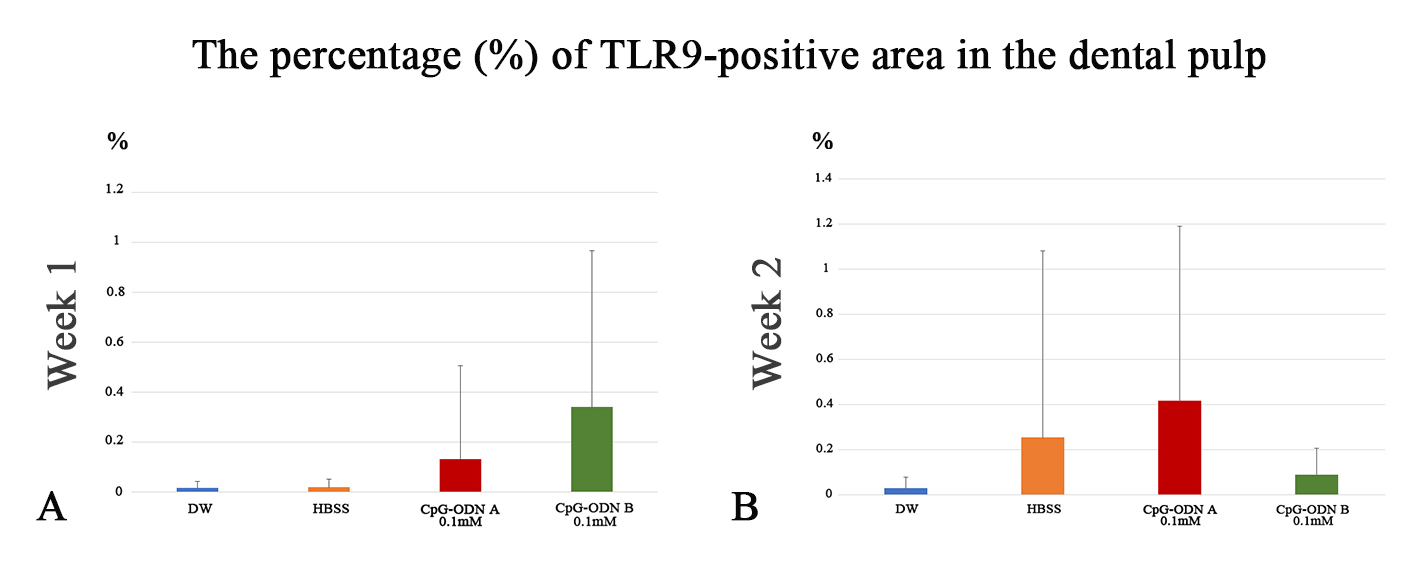

Supplement: Supplementary file 1 [file biomolecules-14-00931-s001.zip › biomolecules-3094805-supplementary/Suppl 5 TLR9 graph plate.jpg]

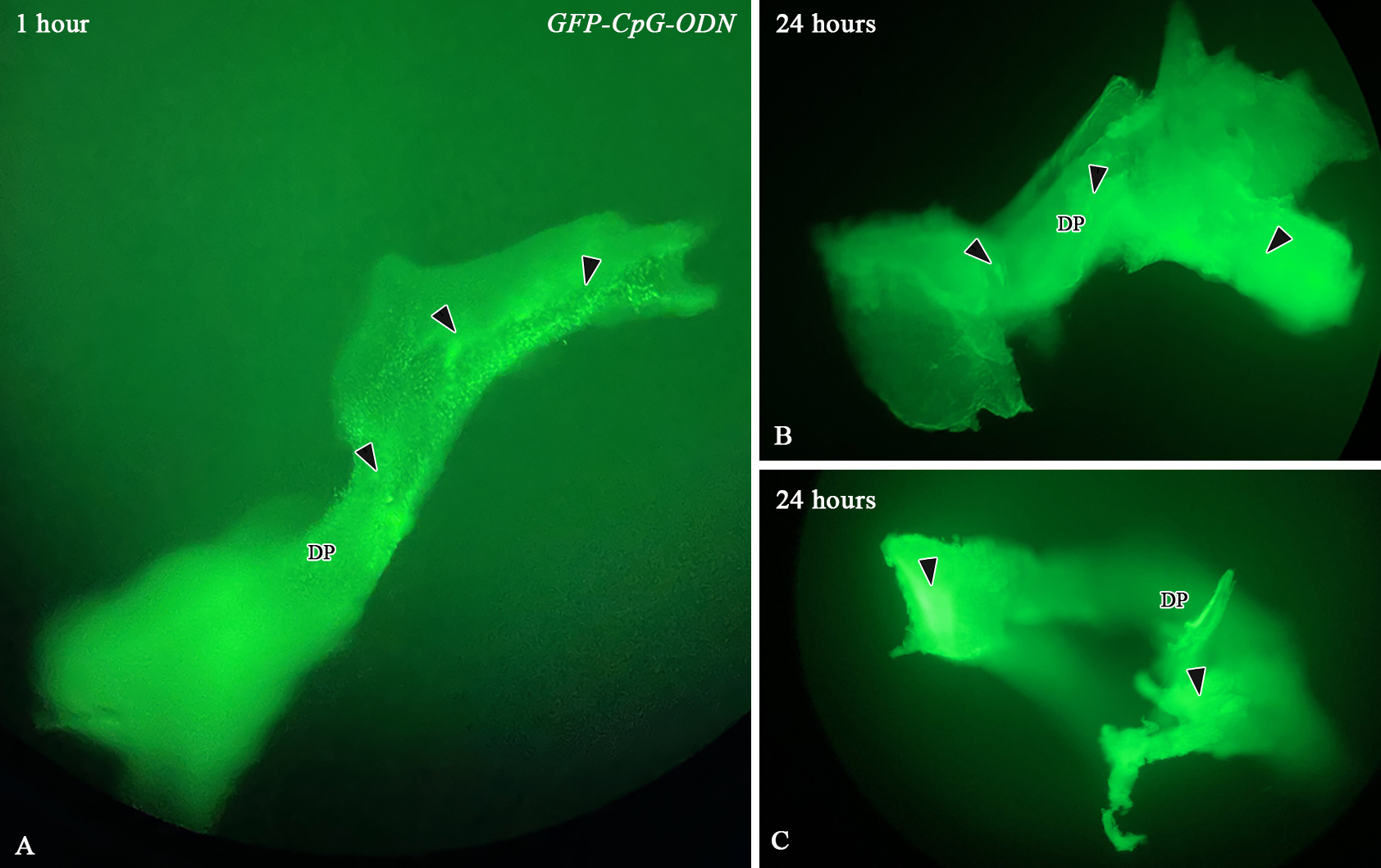

Supplement: Supplementary file 1 [file biomolecules-14-00931-s001.zip › biomolecules-3094805-supplementary/Suppl 6 -GFP-CpG-ODNs.jpg]
